# Supplementary material for: Protocol for genotyping cephalopod sex using a skin swab and quantitative PCR
Source: bioRxiv. 2026 Apr 2:2026.03.31.715692. Preprint. [Version 1] doi: 10.64898/2026.03.31.715692 (PMC13060237; doi:10.64898/2026.03.31.715692)
Supplement: Supplement 1 [file media-1.pdf]

Chromosome	Orthogroup *O. bimaculoides*	Gene ID	Species	Sequence
chr1 (autosome)	OG0008421	pred2_29441.1	*Architeuthis dux*	MTKLEIITPFQLYFNPDLIFKKYHIWRLVTNFMYFGPIGFNFIFNIIFAYRYCRMLEEGSFRGRTSDFFFMFIFGGFVMIFITILIGNIAFLGNAFTIMLVYIWSRRNPYVRMNFFGLLNFQAPYLPWVLLAFSLLLGNSVAVDLMGILVGHLYYYLEDVFPHQVGGFKILRTPQFLKTLMDAAPEDPNYAPLPEDRPGGFNWGNQPQ
		Dopeav2008472m.g	*Doryteuthis pealeii*	MAFQQEYMQMPPITRAYTTACVLTTIAVKLDIITPFQLYFNPDLIFKKYHIWRLVTNFMYFGPIGFNFIFNIIFAYRYCRMLEEGSFRGRTSDFFFMFIFGGFIMILITILIGNIAFLGNAFTIMLVYIWSRRNPYVRMNFFGLLNFQAPYLPWVLLAFSLLLGNSVAVDLMGILVGHLYYYLEDVFPHQVGGFKILRTPQFLKMLMDAAPEDPNYQPLPEDRPGGFNWGNQPQ*
		g1053.t1	*Euprymna scolopes*	MAFQQEYMQMPPITRAYTTACVLTTIAVKLDIITPFQLYFNPDLIFKKYHVWRLVTNFMYFGPIGFNFIFNIIFAYRYCRMLEEGSFRGRTSDFFFMFIFGGFIMIIITILIGNIAFLGNAFTIMLVYIWSRRNPYVRMNFFGLLNFQAPYLPWVLLAFSLLLGNSVAVDLMGILVGHLYYYLEDVFPHQVGGFKILRTPQFLKTLMDAAPEDPNYQPLPEDRPGGFNWGNQGQ
		LOC_00005141-mRNA-1	*Illex illecebrosus*	MAFQQEYMQMPPITRAYTTACVLTTIAVKLEIITPFQLYFNPDLIFKKYHIWRLVTNFMYFGPIGFNFIFNIIFAYRYCRMLEEGSFRGRTSDFFFMFIFGGFIMILITILIGNIAFLGNAFTIMLVYIWSRRNPYVRMNFFGLLNFQAPYLPWVLLAFSLLLGNSVAVDLMGILVGHLYYYLEDVFPHQVGGFKILRTPQFLKTIMDAAPEDPNYAPLPEDRPGGFNWGNQPQ
		obimac_0000979.2	*Octopus bimaculoides*	MAAQTFQQEYMQMPPITRAYTTACVLTTIAVKLDIITPLQLYFNPDLIFKKYQIWRLLTNFMYFGPIGFNFIFNIIFAYRYCRMLEEGSFRGRTSDFFFMFLFGGFIMIINTILIGNIAFLGNAFTIMLVYIWSRRNPYVRMNFFGLLNFQAPYLPWVLLTFSLLLGNSVAVDLMGILVGHLYFYLEDVFPLQMGGFKILRTPRFLKYLMDTTVEDPNYQPFPEDRPGGFDWGNNGN
		XP_029634577.2	*Octopus sinensis*	MRTCQALEARMRINTWHSAMAAQTFQQEYMQMPPITRAYTTACVLTTIAVKLDIITPLQLYFNPDLIFKKYQIWRLLTNFMYFGPIGFNFIFNIIFAYRYCRMLEEGSFRGRTSDFFFMFLFGGFIMIINTILIGNIAFLGNAFTIMLVYIWSRRNPYVRMNFFGLLNFQAPYLPWVLLTFSLLLGNSVAVDLMGILVGHLYFYLEDVFPLQMGGFKILRTPRFLKYLMDTTVEDPNYQPFPEDRPGGFDWGNNGN
		rna-SPHA_31531	*Sepia esculenta*	MPPITRAYTTACVLTTIAVKLDIITPFQLYFNPDLIFKKYHIWRLVTNFMYFGPIGFNFIFNIIFAYRYCRMLEEGSFRGRTSDFFFMFLFGGFIMILITILIGNIAFLGNAFTIMLVYIWSRRNPYVRMNFFGLLNFQAPYLPWVLLAFSLLLGNSVAVDLMGILVGHLYYYLEDVFPHQVGGFKILRTPQFLKTIMDAAPEDPNYQPLPEDRPGGFNWGNQPQ*
		rna-SPHA_31531	*Sepia officinalis*	MPPITRAYTTACVLTTIAVKLDIITPFQLYFNPDLIFKKYHIWRLVTNFMYFGPIGFNFIFNIIFAYRYCRMLEEGSFRGRTSDFFFMFLFGGFIMILITILIGNIAFLGNAFTIMLVYIWSRRNPYVRMNFFGLLNFQAPYLPWVLLAFSLLLGNSVAVDLM
chr1 (autosome)	OG0009041	pred2_18336.1	*Architeuthis dux*	MPHIDNDIKLDFKDVLLRPKRSTIKSRADVDLGREFIFRNSGKTYQGIPIMASNMDTVGTFEMANELSKHRVFTVIHKHYSLDAWKEFAANNPDTLKNIAVSSGMAGGDREKLEAIVNAIPGLHYICLDVANGYSEYFVQFVKDVRKKFPHHVILAGNVVTGEMVEELILSGADIIKVGIGPGSVCTTRKKTGIGYPQLSAVIECADAAHGLGGHIISDGGCTCPGDVAKAFGAGADFVMVGGMLAGHTESGGEMIEKNGKKFKLFYGMSSATAMQKHVGKVAEYRASEGKTVEIAYRGDVQVTMLDILGGIRSTCTYVGASKLKELSRRTTFIRVTQQVNEVFTPFTTAP
		Dopeav2098899m.g	*Doryteuthis pealeii*	MPHIDNDIKLDFKDVLLRPKRSTIKSRADVDLGREFIFRNSGKTYKGIPIMASNMDTVGTFEMANQLAKHGVFTVIHKHYSLDAWKEFAAKNPETLNNIAVSSGMAAGDFEKLEAVINSIPALQYICLDVANGYSEYFVQFVRDVRKRFPKHIILAGNVVTGEMVEELILSGADIIKVGIGPGSVCTTRKKTGIGYPQLSAVIECADAAHGLGGHIISDGGCTCPGDVAKAFGAGADFVMIGGMLAGHTESGGEMIEKNGKKFKLFYGMSSATAMQKHSGKVAEYRASEGKTVEIEYRGDVQGTILDVLGGLRSTCTYVGASKLKELSRRTTFIRVTQQVNEVFSPFTTAH
		g14646.t1	*Euprymna scolopes*	MPHIDNDIKLDFKDVLLRPKRSTIKSRADVDLVREFIFRNSGRTYQGIPVMASNMDTVGTFEMAKQLSKHRVFTVIHKHYSLEAWKEFAAKNPETLKNTAVSSGMAAGDLEKLAAIVNAIPDLHYICLDVANGYSEYFVQFVRDVRKKFPNHVIMAGNVVTGEMVEELILSGADIIKVGIGPGSVCTTRKKTGIGYPQLSAVIECADAAHGLGGHIISDGGCTCPGDVAKAFGAGADFVMVGGMLAGHTESGGDIVEKNGKKFKLFYGMSSATAMQKHSGKVAEYRASEGKTVEIEYRGDVQGTMLDVLGGLRSTCTYVGASKLKELSRRTTFIRVTQQVNEVFSPFTKAP
		LOC_00006323-mRNA-1	*Illex illecebrosus*	MPRIDNDIKLDFKDVLLRPKRSTIKSRADVDLGREFIFRNSGKTYEGIPIMASNMDTVGTFEMANELAKHRVFTVIHKHYSLDAWKEFAANNPDTVKNIAVSSGMAGGDLEKLEAVVNAIPDLNYICLDVANGYSEFFVQFVRDVRKKFPHHVILAGNVVTGEMVEELILSGADIIKVGIGPGSVCTTRKKTGIGYPQLSAVIECADAAHGLGGHIISDGGCTCPGDVAKAFGAGADFVMVGGMLAGHTESGGEMIEKNGKKFKLFYGMSSATAMHKHSGKVAEYRASEGKTVEIAYRGDVQATILDVLGGIRSTCTYVGASKLKELSRRTTFIRVTQQVNEVFSPFTTAP
		obimac_0001039.2	*Octopus bimaculoides*	MPRIDSDIKLDFKDVLLRPKRSTIKSRADVDLTREFIFRNSGQTYNGIPVMASNMDTVGTFEMAITLAKYGLFTTIHKHYSIEAWKEFAANHPDQLSNIAASSGMAAGDLQKLESIIEAVPALRYICLDVANGYSEYFVQFLRDVRKKFPSHVIMAGNVVTGEMVEELILSGADIIKVGIGPGSVCTTRKKTGIGYPQLSAVIECADAAHGLGGHIISDGGCTCPGDVAKAFGAGADFVMIGGMLAGHTESGGEMIEKNGKKFKLFYGMSSATAMQKHAGHVAEYRASEGKTVEIEYRGDASHTVQDILGGIRSTCTYVGASKLKELSRRTTFIRVTQQMNEVFSPFNKSC
		XP_029641704.2	*Octopus sinensis*	MRPEVLGEWVDLTREFIFRNSGQTYNGVPVMASNMDTVGTFEMAITLAKYGLFTTIHKHYSIDAWKEFAAKHPDKLNNIAASSGMAAGDLQKLESIIEAVPDLRYICLDVANGYSEFFVQFLRDVRKKFPSHVIMAGNVVTGEMVEELILSGADIIKVGIGPGSVCTTRKKTGIGYPQLSAVIECADAAHGLGGHIISDGGCTCPGDVAKAFGAGADFVMIGGMLAGHTESGGEMIEKNGKKFKLFYGMSSATAMQKHAGHVAEYRASEGKTVEIEYRGDAAHTVQDILGGIRSTCTYVGASKLKELSRRTTFIRVTQQMNEVFSPFNKSS
		rna-SPHA_21857	*Sepia esculenta*	VDLVREFIFRNSGQTYRGIPIMASNMDTVGTFEMAIQLSKHGVFTVIHKHYSLEAWKEFATKNPETVNNIAVSSGMAGGDLEKLESIVNAIPDLHYICLDVANGYSEYFVQFVRDVRKKFPKHVIMAGNVVTGEMVEELILSGADIIKVGIGPGSVCTTRKKTGIGYPQLSAVIECADAAHGLGGHIISDGGCTCPGDVAKAFGAGADFVMVGGMLAGHTESGGEMIERNGKKFKLFYGMSSATAMKKHAGKVAEYRASEGKSVEIEYRGDVQGTILDILGGIRSTCTYVGASKLKELSRRTTFIRVTQQLNEVFSPFTKAP
		rna-SPHA_21857	*Sepia officinalis*	MPHIDNDIKLDFKDVLLRPKRSTIKSRADVDLVREFLFRNSGQTYRGIPIMASNMDTVGTFEMAIQLSKHGVFTTIHKHYSLEAWKEFATKNPETVNAGNVVTGEMVEELILSGADIIKVGIGPGSVCTTRKKTGIGYPQLSAVIECADAAHGLGGHIISDGGCTCPGDVAKAFGAGADFVMVGGMLAGHTESGGEMIERNGKKFKLFYGMSSATAMKKHAGKVAEYRASEGKSVEIEYRGDVQGTILDVLGGIRSTCTYVGASKLKELSRRTTFIRVTQQLNEVFSPFTTAP

chr1 (autosome)	OG0009283	pred2_28664.1	*Architeuthis dux*	MSYGRRGPSELDLEGMVSLKVDNLTYRTTPEDLRRAFEKYGDVGDVYIPRDRFTRESRGFAFVRYHDKRDADDAMDAMDGAIMDGRELRVQMARYGRPADPYRRGPPPRRFGGGGGGGGGGGGGGGGGGGGGYSSYRSRRRSYSRSRSRSRSRSSRRRRRSYSRSRSRSRSRSYKSKSRSRSRSPSYSRSSRSKHYSRSPTPPSKGSGSKKRSSGGSRRSTRSRSPSRDDRKNSRSRSRSPSPARSSEGGRGESQGRGSGGPTGGGGASAGASGSGSSLAPAASTAGGGAGGAPRENSSKSRSRSRSRSRSRSRSRSRSRSNSRSPRYASVSPEGSPEANSNNHKDQQQADEED
		Dopeav2005453m.g	*Doryteuthis pealeii*	MSYGRRGPSELDLEGMVSLKVDNLTYRTTPEDLRRAFEKYGDVGDVYIPRDRFTRESRGFAFVRYHDKRDADDAMDAMDGAIMDGRELRVQMARYGRPADPYRRGPPPRRFGGGGGGGGGYSSYRSRRRSYSRSRSRSRSRSSRRRRRSYSRSRSRSRSRSYKSRSRSRSPSYSRSSRSKHYSRSPTPPSKGSGSKKRSSGGSRRSTRSRSPSRDDRKSRSRSRSPSPARSSEGGRGGSQGRSSGPAGGVGSAGGSGSSGLAPAAGTASGGPPRENSKSRSRSRSRSRSRSRSRSRSRSHSRSRSRSPRSRSCSPEGSPDANSNNHKDKQQVDEED
		g752.t1	*Euprymna scolopes*	MSYGRRGPSELDLEGMVSLKVDNLTYRTTPEDLRRAFEKYGDVGDVYIPRDRFTRESRGFAFVRYHDKRDADDAMDAMDGAIMDGRELRVQMARYGRPADPYRRGPPPRRFGGGGGGGGGGGGGGGYSSYRSRRRSYSRSRSRSRSRSSRRRRRSYTRSRSRSRSRSYKSRSRSRSPSYSRSSRSKHYSRSPTPPSKGSGSKKRSSGGSRRSTRSRSPSRDDRKSRSRSRSASPARSSEGGRGGSQTRGSSGPPASSGATASASGNLTPSGSATASGGAPRENSSKSRSRSRSRSRSRSRSRSPHSRSRSRSRSRSCSPQGSPDANSNNHKDKQEVDEED
		LOC_00005496-mRNA-1	*Illex illecebrosus*	MSYGRRGPSELDLEGMVSLKVDNLTYRTTPEDLRRAFEKYGDVGDVYIPRDRFTRESRGFAFVRYHDKRDADDAMDAMDGAIMDGRELRVQMARYGRPADPYRRGPPPRRFGGGGGGGGGGGGGGGGGGGGGYSSYRSRRRSYSRSRSRSRSRSSRRRRRSYTRSRSRSRSRSYKSRSRSRSPSYSRSSRSKHYSRSPTPPSKGSGSKKRSSGGSRRSTRSRSPSREERKTSRSRSRSPSPARSSEGGRAGSQGRGSSGPAAGAGAGGSAGASGSSSSLVPAAGTTSGGAGAARENSSKSRSRSRSRSRSRSRSRSRSRSRSHSRSPRSRSVSPEGSPEANSNNHKDQQQADEED
		obimac_0002028.1	*Octopus bimaculoides*	MSYVRRGPSELDLEGMVSLKVDNLTYRTTPEDLRRAFEKYGDVGDVYIPRDRFTRESRGFAFVRYHDKRDADDAMDAMDGAMMDGRELRVQMARYGRPADPYRRGPPPRRFGGGGYSSYRSRRRSYTRSRSRSRSRSSRRRRRSYSRSRSRSRSRSYKSRSRSRSASYSRSSRSKHYSRSPSPSSKGKKRSSASSRRSTRSRSASRERKSHSRSRSPVSPRRSPDRCSEGRDGSESGAGANAGGGGGGGSSGSVPRENSKSRSRSRTPHSRSCSPEALSDRGSNDHNDLEQQVEEED
		XP_029633039.1	*Octopus sinensis*	MSYVRRGPSELDLEGMVSLKVDNLTYRTTPEDLRRAFEKYGDVGDVYIPRDRFTRESRGFAFVRYHDKRDADDAMDAMDGAMMDGRELRVQMARYGRPADPYRRGPPPRRFGGGGYSSYRSRRRSYTRSRSRSRSRSSRRRRRSYSRSRSRSRSRSYKSRSRSRSASYSRSSRSKHYSRSPSPSSKGKKRSSASSRRSTRSRSASRERKSHSRSRSPVSPRRSPDRCSEGRDGSESGAGANAGGGGGGSSGSVPRENSKSRSRSRTPHSRSCSPEALSDRGSNDHNDLEQQVEEED
		rna-SPHA_39875	*Sepia esculenta*	MSYGRRGPSELDLEGMVSLKVDNLTYRTTPEDLRRAFEKYGDVGDVYIPRDRFTRESRGFAFVRYHDKRDADDAMDAMDGAVMDGRELRVQMARYGRPADPYRRGPPPRRFGGGGGGGGGGGYSSYRSRRRSYSRSRSRSRSRSSRRRRRSYTRSRSRSRSRSYKSRSRSRSPSYSRSSRSKHYSRSPTPPSKGSGSKKRSSGGSRRSTRSRSPSRDDRKSRSRSRSPGSPARSSEGGRGGSQGRSSGPAGGGGSTGGSGSGTLAPAAGTASGGPPRENSSKSRSRSRSRSRSRSRSRSRSRSRSRSRSPRSRSCSPEGSPDANSNNHKDKQQIDEED
		rna-SPHA_39875	*Sepia officinalis*	MSYGRRGPSELDLEGMVSLKVDNLTYRTTPEDLRRAFEKYGDVGDVYIPRDRFTRESRGFAFVRYHDKRDADDAMDAMDGAVMDGRELRVQMARYGRPADPYRRGPPPRRFGGGGGGGGGGGGGYSSYRSRRRSYSRSRSRSRSRSSRRRRRSYTRSRSRSRSRSYKSRSRSRSPSYSRSSRSKHYSRSPTPPSKGSGSKKRSSGGSRRSTRSRSPSRDDRKSRSRSRSPGSPARSSEGGRGGSQGRSSGPVGGGSSAGGSGSGTLAPAAGTASGGAPRENSSKSRSRSRSRSRSRSRSRSRSRSRSRSRSPRSRSCSPEGSPDANSNNHKDKQQIDEED
chr1 (autosome)	OG0009320	pred2_29997.1	*Architeuthis dux*	MLPGVGVFGTTATIQSFVPILKVCGFPVVALWGSENLDAQELATQLDIPFSTRKVDDVLLRKDVDLVVIGCPPHSQWFIAVKALGIGKHVLCSAPSGPMQLNAQHMVKAARYYPRLMSLMCYGLRFLPTIVKMKKMIEDGCLGNITICEVKVHYGGLPKEKYDWMCDEGMGGGVLNTIGSNIIDVITFLTKERAVRVHGMLKTYTKQTQNIKGIREITSDDFCTFQMELDKGACVTVTLNSHIPGQFVQEIVICGQKGRLIARGSDLYEQRLNATRESLIHFDPIKEEERYGISPKARTEIPSPYLKGLIHMIEAVKDAFEKEEERQNWQAEPVASASNFEDALYVQTVVDAIRKSNKTKQWTKVDVSVEEPEPNSNNMLSDQMRRSTFSLQ
		Dopeav2006705m.g	*Doryteuthis pealeii*	MLPGVGVFGTTATIQSFVPILKVCGFPVVALWGRENLDAQELATQLDIPFSTRKVDDVLLRKDVDLVVIGCPPHSQCFIAVKALGIGKHVLCSAPSGPMQLNVQRMVKAARYYPRLMSLMCYGLRFLPTIVKMKKMIEEGCLGSITICEVKVHYGGLPKEKYDWMCDEGMGGGVLNTIGSNIIDVITFLTKERAVRVHGMLKTYTKQTQNIKGIREITSDDFCTFQMELDKGACVTVTLNSHIPGEFVQEIVICGQKGRLIARGSDLYEQRLNATRESLIHFDAIKEEEKYGISPKARTEIPIPYLKGLIHMIEAVKDAFEKEEERQNWQGEPVASASNFEDALYVQTVVDAIRKSNKTKQWTKVDVSVEEPEPNSNNMLSDQMRRSTFSLQ
		g828.t1	*Euprymna scolopes*	MLPGVGVFGTTATIQSFVPILKVCGFPVVALWGSENLDAQELATQLDIPFSTRKVDDVLLRKDVDLVVIGCPPHSQWFIAVKALGIGKHVLCSAPSGPMQLNAQHMVKAARYYPRLMSLMCYGLRFLPTIIKMKTMIEEGCLGSITICEVKVHYGGLPKEKYDWMCDEGMGGGVLNTIGSNIIDVITFLTKERALRVHGMLKTYTKQTQNIKGIREITSDDFCTFQMELDKGACVTVTLNSHIPGQFVQEIVICGQKGRLIARGSDLYEQRLNATRESLIHFDPIKEEERYGISPKARTEIPSPYLKGLIHMIEAVKDAFEKEEERQNWQAEPVASASNFEDALYVQTVVDAIRKSNKTKQWTKVDVSVEEPEPNSNNMLSDQMRRSTFSLQ
		LOC_00005427-mRNA-1	*Illex illecebrosus*	MLPGVGVFGTTATIQSFVPILKVCGFPVVALWGSENLDAQELATQLDIPFSTRKVDDVLLRKDVDLVVIGCPPHSQWFIAVKALGIGKHVLCSAPSGPMQLNAQHMVKAARYYPRLMSLMCYGLRFLPTIIKMKKMIEEGCLGSITICEVKVHYGGLPKEKYDWMCDEGMGGGVLNTIGSNIIDVITFLTKERAVRVHGMLKTYTKQTQNIKGIREITSDDFCTFQMELDKGACVTVTLNSHIPGQFVQEIVICGQKGRLIARGSDLYEQRLNATRESLIHFDPIKEEERYGISPKARTEIPSPYLKGLIHMIEAVKDAFEKEEERQNWQTEPVASASNFEDALYVQTVVDAIRKSNKTKQWTKVDVSVEETNSNNMLSDQMRRSTFSLQ
		obimac_0001416.1	*Octopus bimaculoides*	MLPGVGVFGTTATIQSFVPILKVCGFPVVALWGSENLDARTLAAELDIPFSTHKVDDVLLRKDVDLVVIGCPPHSQSYIAVKALGIGKHVLCSSPSGPMQLNALLMVKAARYYPRLMSLMCYGLRFLPTIVKMKKIIEDGCLGNITICEVKVHYGGLPKEKYDWMCDEGMGGGVLNTFGSNIIDVITFLTSERAVRVHGMLKTYTKQTQNIKGIREITSDDFCTFQMELDKGACVNVTLNSHITGQFVQEILVCGRKGRLIARGSDLYEQRLNATRESLIHFDPIKEEERYGISPKARTEIPSPYLKGLIHMIEAVKDAFEKEEERQNWHPEPVASASNFEDALYVQAVVDAIRKSNKTKQWTKVDVSVNEPEPEPNSNSMSLDYVRRSTIFLH

		XP_029644474.1	*Octopus sinensis*	MLPGVGVFGTTATIQSFVPILKVCGFPVVALWGSENLDARTLAAELDIPFSTHKVDDVLLRKDVDLVVIGCPPHSQSYIAVKALGIGKHVLCSSPSGPMQLNALLMVKAARYYPRLMSLMCYGLRFLPTIVKMKKIIEEGCLGNITICEVKVHYGGLPKEKYDWMCDEGMGGGVLNTFGSNIIDVITFLTSERAVRVHGMLKTYTKQTQNIKGIREITSDDFCTFQMELDKGACVNVTLNSHITGQFVQEILVCGRKGRLIARGSDLYEQRLNATRESLIHFDPIKEEERYGISPKARTEIPSPYLKGLIHMIEAVKDAFEKEEERQNWHPEPVASASNFEDALYVQAVVDAIRKSNKTKQWTKVDVSVNEPEPEPNSNSMSLDYVRRGTIVLH
		rna-SPHA_69492	*Sepia esculenta*	MLPGVGVFGTTATIQSFVPILKVCGFPVVALWGSENLDAQELATQLNIPFSTRKVDDVLLRKDVDLVVIGCPPHSQWFIAVKALGIGKHVLCSAPSGPMQLNAQHMVQAARYYPRLMSLMCYGLRFLPTIVKMKKMIEEGYLGNITICEVKVHYGGLPKEKYDWMCDEGMGGGVLNTIGSNIIDVITFLTKERAVRVHGMLKTYTKQTQNIKGIREITSDDFCTFQMELDKGACVTVTLNSHIPGQFVQEIVICGQKGRLIARGSDLYEQRLNATRESLIHFDPIKEEERYGISPKARTEIPSPYLKGLIHMIEAVKDAFEKEEERQNWQAEPVASASNFEDALYVQTVVDAIRKSNKTKQWTKVDVSVEEPEPNSNMLSDQMRRSTFSLQ
		rna-SPHA_69492	*Sepia officinalis*	MLPGVGVFGTTATIQSFVPILKVCGFPVVALWGSENLDAQELATQLDIPFSTRKVDDVLLRKDVDLVVIGCPPHSQWFIAVKALGIGKHVLCSAPSGPMQLNAQHMVQAARYYPRLMSLMCYGLRFLPTIVKMKKMIEEGYLGNITICEVKVHYGGLPKEKYDWMCDEGMGGGVLNTIGSNIIDVITFLTKERAVRVHGMLKTYTKQTQNIKGIREITSDDFCTFQMELDKGACVTVTLNSHIPGQFVQEIVICGQKGRLIARGSDLYEQRLNATRESLIHFDPIKEEERYGISPKARAEIPSPYLKGLIHMIEAVKDAFEKEEERQNWQAEPVASASNFEDALYVQTVVDAIRKSNKTKQWTKVDVSVEEPEPNSNMLSDQMRRSTFSLQ
chrZ (sex)	OG0008779	pred2_09431.1	*Architeuthis dux*	MNGELKEKFEPHKSSDFLERISQKSISEDIKTEIQPTGNLKFRSEQLAYTRSGDRRNTADIAPPFQPSQKSSPAGSFKDFHKNVDMFSQGDNKIHIHGMTRDCHQAPFD
		Dopeav2130673m.g	*Doryteuthis pealeii*	MKLLSNICLHLCRNKQYLFRRNLLSVVPMTRRHNHQENGKRPSSVRIGCGSGFWGDSSIAAQQLIHYGKIDFLVFDYLSEITMSLLTAMKQKNPDMGYAPDFVHFSMVPHLGTIKEKGIKVVSNGGGVNPHACAKLLGDFCKKQNVDLNIAVVTGDDLMIELKKIKQLNLKDMSSGMEFPKTVHSMNAYLGAGPIARALDLGADIVVTGRCVDSALVLGPLLHTFKYKKNDFDQLASGSLAGHIVECGAQATGGNFSDWHTVQNWDTIGFPIVEFSDDGSFIVTKPQSSGGCVTRATVCEQMLYEIGDPKRYQLPDVTADFSQVQINEIEGMTDAVLVKGAKGTPPSDSYKITATYADGFRATAVACVGGPRSEEKAIKTADAILERCRKMFKMLNLGDFTKVHVEILGSEPNSSGSQIIPRQLGLWLAVHHSNKKALEFFAREIAPAGTGMAPGLTGIVGGRPRVSPVLKLFSFLYPKDNLNVEIFINGEQKEKFEPFKNSDIPQTIKSEEPTTEDAKSENLPVGNFNFRLEDLAYTRSGDKGNSANIGVIARDPSYLPYLRQALTEEAMEQYFSYLFEDNNGVRVTRYDVPGIDGINFVLHNSLGGGGIASLRSDPQVRKENIYYHYLEEKCNSQGLVSDL
		g22135.t1	*Euprymna scolopes*	MTTRLNHHGKPLKSVRIGCGSGFWGDSSIAAEQLIHQGKIDFLVFDYLSEITMSLLTAVKNKHPKLGYTPDFVQYSLLPHLKAIKKKGIKVISNGGGVNPHACGNLIMDFCKKNDIDLNVAVVTGDDLMSELKKMEQFGPKEMLSGMEFPKTVNSMNAYLGAGPIARALDLGADIVVTGRCVDSALVVGPLLHKFKYKMSDFDLLASASVAGHIVECGAQATGGNHSDWKIVKDWANIGFPIVDFSKDGSFIITKPDSTGGCVTCGTVGEQMLYEIDDPQKYLLPDVTVDFSRIQIREIEGRTDAIQVVGAKGYPPTKDYKVTATYADGYRATAVACVGGPRSEEKARKTANALLERCRSWFKRLNLGDFSKVNIEILGSETEQPFSQVVPRQLALWLAVNHPKKNALEFFAREIASAGTGMAPGLTGIVGGRPRISPVLKLFSFLYPKDSINVEIFINGEMKEIFYQDLDLCSDSSEIVKPVCEDIHTETLLTGNFNYTLQDLAYTRSGDKGNSANIGVIARDPSYLPYIKRALTAEAVKEFFMYAFEDTEKARVTRYNVPGIHGLNFVLHDSLGGGGIASLRSDPQGKAYGQILMDFPIKNVPKLLP
		LOC_00003890-mRNA-1	*Illex illecebrosus*	MRLLKSTCLHLHRSRQCLFSSRNLLSVVPMTKRYNNLQVSSLSGERPNKAVRIGCASGFWGDSSVAAQQLIHHGKIDYLVFDYLSEITMSLLTAAKQRNPDMGYAPDFIHFSVAPHLQMIKDKGIKVVSNGGGVNPHACAKLLKDFCEKAKIDLNIAVVTGDDLMPEIKKIKELSPKDMSSGMEFPKAVHSMNTYLGAGPIARALDLGADIVVTGRCVDSALVLGPLLHTFKYKNNDFDQLAAGSLAGHIVECGAQATGGNFSDWYTVENWDSIGFPIVEFSEEGSFIVTKPESTGGCVTRATVSEQMLYEIGDPKRYLLPDVTADFSQVHIKEIEGMSDAVLVQGAKGTPPSNSYKVTATYADGFRATAVACIGGPRSEEKGLKTANSIFEKCRKMFKKMNMADFSKVHVEILGSESQTSPSQSTPRQVGLWMAVHHYNKKALEFFAREIAPAGTGMAPGLTGIVGGRPRVSPVLKLFSFLYPKDDINVEIFMNGELKERFEQFETTEFPETVDQKPTEDVKNEKLPRGNFQFRLEDLAYTRSGDKGNSANIGVIARDPSYLPYLREALTEEAVREYFSYLFEDSKSGRVTRYDVPGVHGLNFVLHDSLGGGGIASLRSDPQGKAFGQILMDFPITGVPNLCNKK
		obimac_0008956.1	*Octopus bimaculoides*	MSASRMSSKLFLNNRYSLKKFVSFNRQYCYKIPNEHDVVRIGCASGFWGDTAVAAPQLIHHGKIDFLVFDYLSEITMSLLTAAKQKNPAMGYAPDFIHFSLAPYLKTIKEKKIRVVSNAGGINPLSCAEVLKKLSKEAGVDMNIAVVTGDDLMPKVKEISQSNIQEMSSGMKIPKTIHSMNAYLGAGPIARALDLGADIVVTGRCVDSALVLGPLIHAFKYNNSNFDQLASASLAGHIIECGAQATGGVFTDWQTVHNWDNIGFPIVEFSANGSFSVSKPPSTGGLVSKGTVCEQLLYEIGDPKNYILPDIICDFSQVQVFESEKDSVQVKGALGKPPTNDYKVTATYADGYRVTAVTCIGGPQAEEKAKKTTDAILKRCRNIFKQLKLGDFIRVNVEILGFESKNENKEEPRQLAVWMAAHHPKKQALEILSREIAPAGTGMAPGLTAIAGGRPRVSPVLKLFSFLYPKDELKVQIYMNGELKESYQPSEYSPTLKENVSESSANSEESNLPTGNCEFYLKDLAYTRSGDKGNSANIGVIARHPSYLPYLRAALTESAVEKYFSSLFEDDDDGKRVTRYDVPGINAMNFVLHNCLGGGGIASLQSDPQGKALGQHLLNFKITNVPDLLSKIF
		XP_029648810.2	*Octopus sinensis*	MSASRMSAKLLLNNRYSLKKFVCFNRQYCYKIPNENDVVRIGCASGFWGDTAVAAPQLIHHGKIDFLVFDYLSEITMSLLTAAKQKNPAMGYAPDFIHFSLAPYLKTIKEKKIRVVSNAGGINPLSCAEVLKKLSQEAGVDMNIAVVTGDNLMPKVKEISQSNIQEMSSGMKIPKTIHSMNAYLGAGPIARALDLGADIVVTGRCVDSALVLGPLIHAFKYNNSNFDQLASASLAGHIIECGAQATGGVFTDWQTVHNWDNIGFPIVEFSANGSFSVSKPPSTGGLVSKGTVCEQLLYEIGDPKNYILPDIICDFSQVQVSESGKDNVQVKGALGKPPTNDYKVTATYADGYRVTAVTCIGGPQAEEKAKKTMDAILKRCRNIFKQLKLGDFTRVNIEILGSESKNENNKEPRQLAVWMAAHHPKKQALEILSREIAPAGTGMAPGLTAIAGGRPRVSPVLKLFSFLYPKDELQVQIYMNGELKESYQPSEYSPTSKENVSESSANSEESNLPTGNCEFYLKDLAYTRSGDKGNSVNIGVVARHPSYLPYLRAALTESAVEKYFSFLFEDDDDGERVTRYDVPGINAMNFVLHNCLGGGGIASLRSDPQGKENLINRSIERLIRCNHRFKYTFVQYNRND
		rna-SPHA_78642	*Sepia esculenta*	MNTLVLIVDDERSGRTIVVVALAVDNVCLSATETAVASSSFSAIAALGEYKFIFFFQLLPKKSKEQ*DY*KIFVYV*TGTGTAWKWEISQNCENWMRKWFLG*QLCCGSATYPSWQN*FSCF*LFI*NHNVSSHCC*TKES*YGLCTRLCTFFFGTLP*NYKR*RYQGCQQCWRCESTCLCQITKRVLHEKEC*PQNCCSNRR*FDA*D*ENETIIS*RHVFWYGIS*NCS*YECLSWCRSY*KGS*PWC*YCCDWQMCGQCFGPWTAPSYFQIQKE*F*PTCIW*SCWSHSRVRSSSYWR*L**LAYS*KLGYNWFPNCGIFRGWLFPCYQTPINWWLCHKSHCL*TNVI*NWRS*EISIT*RDS*F*PSADKGN*RNEGYGPSTRC*RNSTIQ*L*GYCNLC*WFSSYSSCMCWGTSQ*RESNKNS*CYFRKMQKNIQKAKFG*FY*SPCGTLGIRTIYILPSDNSTTIRTLVSSST***NSPGDFCSRDCTSWNRNGTWINRNSWRKTSSISCTEAFFIFVS*RQYHRKSFHKW*AKRKV*VTEKFRIF*NSKPEDNF**QED*NTSRWKLYVQIGRACLHSKW**RKYSKYRGHSTRSFLSSLPQESSYRGSHQRILQLFV*R*QKSTSDKV*CTRYTWNKFCFTQLTWWGWNCISQE*SSGKSPRPDSDGFSNYMCS*PV**V

		rna-SPHA_78642	*Sepia officinalis*	LVLFVDDESGGTTVVVALVVDNVCLSATETAVASSSFSAIAALGEYKFIFFFFFNYCRKK*RTMRLLKNICLRLNWNRHCLKVENVPKL*ELDAEVVFGVTALLRIEKMKQLSPKDMSSGMEFPKTVHSMNVYLGSNTKRMILTYLHLVVLLVT**SVELKLPEVTLVTGIQLRIEGMKDMVLVQGAKGTQPSNDYKVTATYADGFRATAVACVGGPRSEEKAMKTADAILERCRKIFKKLNLADFTKVNVELLGSEPDTTCPQTIPRQLGLWLAVQHKNKTALDFFAREIAPAGTGMAPGLTGIVGGRPRVSPVLKLFSFLYPKDNITGSLHEIRLIFLTSRKLLQRKPSKNTSAICLKITEEHK**VMMYQVYME*ILFYTTHLVGVELLLSGLIL
chrZ (sex)	OG0008809	pred2_10461.1	*Architeuthis dux*	MTSKSKLIKVVLLGDGGVGKSSLMNRFVSNKFDTQSFHTIGVEFLNKELTLGTDSYTLQIWDTAGQERFKSLRTPFYRGADCCLLTFAVDDKRSFENISMWRKEFAYYADIADDNVSSFPFVIIGNKVDVFERQVDTAEAAEWCEQSGHLPYFETSAKDATNVESAFVAAVKRLREFEEASTTNDLKDSSQGNTVDLSKKRDQTSNSGCCN
		Dopeav2114893m.g	*Doryteuthis pealeii*	MMSSKSKLIKVVLLGDGGVGKSSLMNRFVSNKFDTQSFHTIGVEFLNKELSLGNDSYTLQIWDTAGQERFKSLRTPFYRGADCCLLTFAVDDKRSFENISMWRKEFAYYADIADDNVSSFPFVIIGNKVDVSDRQVTTGEAAEWCEQSGNLPYFETSAKDATNVEAAFVAAVKRLREFEEASTNNDLKDSTQGNTVDLSKKRDQISNTGCCN*
		g16856.t1	*Euprymna scolopes*	MSTKSKLIKVVLLGDGGVGKSSLMNRFVSNKFDTQSFHTIGVEFLNKEITLGNDTYTLQIWDTAGQERFKSLRTPFYRGADCCLLTFAVDDKRSFENISMWRKEFAYYADIADDNVGTYPFVIIGNKIDVSERQVTTAESSEWCEQNGKLPYFETSAKDATNVEAAFVAAVKRLRDFEEASTTNDLKDSSQGNTVDLSKKRDQVNSSGGCCN
		LOC_00002564-mRNA-1	*Illex illecebrosus*	MTSKSKLIKVVLLGDGGVGKSSLMNRFVSNKFDTQSFHTIGVEFLNKELTLGNDSYTLQIWDTAGQERFKSLRTPFYRGADCCLLTFAVDDKRSFENISMWRKEFAYYADIADDNVSSYPFVIIGNKVDVVERRVDTAEAAEWCEQNGKLPYFETSAKDATNVESAFVAAVKRLREFEEASTTNDLKDSSQGNTVDLSKKRDQSSSTGCCN
		obimac_0008634.1	*Octopus bimaculoides*	MMGSKSKLLKVVLLGDGGVGKSSLMNRFVSNKFDTQSFHTIGVEFLNKEISIGSESYTMQIWDTAGQERFKSLRTPFYRGADCCLLTYAVDDVKSFENVSMWKKEFLYYADIDDESTFPFVVIGNKIDVGQRLVSYNTASEWCEKNGHVPYFETSAKDSTNVEVAFTAAVKRLKDLEDKCAEVKPSHGNTVDLNKKKESQSTGCCN
		XP_036367568.1	*Octopus sinensis*	MQAEKLVQQNLVAFILKHLFGCVYEHKQMGSKSKLLKVVLLGDGGVGKSSLMNRFVSNKFDTQSFHTIGVEFLNKEISIGSESYTMQIWDTAGQERFKSLRTPFYRGADCCLLTYAVDDVKSFENVSMWKKEFLYYADIDDESTFPFVVIGNKIDVGQRLVSYNTASEWCEKNGHVPYFETSAKDSTNVEVAFTAAVKRLKDLEDKCAEVKPSHGNTVDLNKKKESQSTGCCN
		rna-SPHA_58078	*Sepia esculenta*	MTYLMTSLIPPFWNANFYVILLNRFDEKGRFPFNTMSSKSKLIKVVLLGDGGVGKSSLMNRFVSNKFDTQSFHTIGVEFLNKELTLGNDSYTLQIWDTAGQERFKSLRTPFYRGADCCLLTFAVDDKRSFENISMWRKEFAYYADIADDNVSSFPFVIIGNKVDVFERQVTTGEATEWCDQNGKLPYFETSAKDATNVEAAFVAAVKRLREFEEAGTTHDLKDSTQGNTVDLSKKRDQISNSGCCN
		rna-SPHA_58078	*Sepia officinalis*	MTSLMTSLMPPFWNANFYVILLNRFDEKGRFPFNTMSSKSKLIKVVLLGDGGVGKSSLMNRFVSNKFDTQSFHTIGVEFLNKELSLGNDSYTLQIWDTAGQERFKSLRTPFYRGADCCLLTFAVDDKRSFENISMWRKEFAYYADIADDNVSSFPFVIIGNKVDVSERQVTTGEATEWCDQSGKLPYFETSAKDATNVEAAFVAAVKRLREFEEAGTTHDLKDSTQGNTVDLSKKRDQISNSGCCN
chrZ (sex)	OG0009225	pred2_26014.1	*Architeuthis dux*	MLQQQPNSLEPVSHFATKAAMPSLSPEWRFLPELAPTNIESNNKESGKESYFPDAGGIKKYNREFLLELRHTRASLLMPQCLPDLPKELLKMPYPSKMFNLSCSDPFCKGFKDRRPAEKSRSDEGKEVNCLTDTLCNKTTERYKPLLSEIRCIQIDTESQLKAIIDLLFEKASNYPILGISYAYLCRSLSLIRVPSATRQGETVNFNKLLNRRCQIELEKIKQEDEAICQTKQMISAENDEARIMKLQMKLGELTSNSNKRTLGNMKLIGEFVKLRILKESIIIQFVCSLLSSRTEARIECLCVLLKTVGMELEHNSNRQDKKKIEDCFTEMKKMVSQGTCSPRVKAILSSIIRLRENKWVF
		Dopeav2060590m.g	*Doryteuthis pealeii*	MLHQQPNSLESVNNFTTKAVISNLSPEWKFSPELATTNMESNNNNKETGQESCFPDAGGIKKYNREFLLQLRHTRASLLMPPCLPDLPKELLKMPYSSKMFNLSCSDPFYKGLKDRSPVEKSRSDESKESNCLMDTFCKIRCIQIDTESQLKAIIDLLFEKASNYPILGISYAYLCRSLSLIRVPSATRQGETVNFNKLLNRRCQIELEKIKQEDEAINQTKQMVRSAESDEPRIIKLQKKLGELTSASKKRTLGNMKLIGEFFKLRILKENIIIQFVCSLVSSRSEDRIECLCVLLKSVGLELDRSSNRQDKKKMEDCYTEMKKIISQGTCSPRVKAMLSSIIRLRENKWVF*
		cluster_18938	*Euprymna scolopes*	MMSSLAPEWRFLSKSAPTNIEEPRQESCFQSATGIKKYSREFLLQLRHTRASLLMPACLPDLPRELIKMQYSSETFNLSCSDPFFKSLKDRRPSEESRNDKNNETKKPLLSEIRCIQFDTESQLKAIIDLLFEKASKNPILGISYAYLCRSLSLVRVPSSTRQGETVNFNKLLNRRCQIELEKIKQANEAINQAKQMIRSAENGEARDIQLQKKLDELMSTSRKRTMGIMKMIGEFFKMRILNENIITEFVCSLLSPRTEDRVECLCDLLKTVGKELEQNSNRQEKKKMEDCFSEIKKTVSQAGEVHQANYNTTTGLQRIPGGPPPLCQKQLIACADVIRRPSTFARLHRLTGTNQQNEQKES
		LOC_00011915-mRNA-1	*Illex illecebrosus*	MLQQQPNPLESVSSFTTKAAMPSLSPEWRFSPESAPTNIEPNNKESEKESYFPDAGGIKKYNREFLLQLRHTRASLLMPPCLPDLPRELLKIPYPSKMFNLSCSDPFFKGLKDRRPAEKSRSDEGKEVNCLTDTICSKTTERYKPLLSEIRCIQIDTESQLKAIIDLLFEKASNYPILGISYAYLCRSLSLIRVPSATRQGETVNFNKLLNRRCQIELEKIKQEDEAISQTRQFIRNAENDEARVMKLQKKLAELTTSSKKRTLGNMKLIGEFFKLRILKENIVIQFVCGLLSSRTEDRIECLCILLKTVGTELERNSNRQDKKKMEDCFTEMKKVVSQGTCSPRVKTMLSSIIRLRENKWVF
		obimac_0009088.1	*Octopus bimaculoides*	MPFSNSPSENAYSYPNSNVSSTEETQEATQRTPLPSQIRLKYDRDFILKLQYKEASLTKPVGLPDLPEILFKKPQIPKQLKYNKIRRDIIPFKCSKNMKSVDEYVNDSEDEELNTKMKNIASRLAPEKYKSVLSQIRDIQIDKESKLVAIMNLLFEKATSDPMLSTAYAYVCRCLTLKRVPSIHCHKEVVNVHKVLNKKCQKEFEKYQVEEAMLNKKLQQIEDTDNNIEKLKFQKELVKDIEIFKRKSQANVRFLCELFKLGVLRENTMQKYIKKMLQSLSENSLESVCLLFNTIGERLDTGKKFKMDVYFIQLKSIAEENSTPNRLKVMIENLISLRENQWNQKEAENHTKQKGMTSVKDSMKEETLKVMANSYYENKVKDIYQNGDGSVASNMGDIFSSDPGTKTSSSYQQPQQQQQQQQQHIKEEYVKPGYMKIFDIDAQNKCMDLTQLERKYCNLVYDGTTRASARGDVMVPNCSPNIIRETKEAV
		XP_036367469.1	*Octopus sinensis*	MPFSNSPSENAYSYPNSNVSSTEETQEATQRTPLPSQIRLKYDRDFILKLQYKEASLTKPVGLPDLPEILFKKPQIPKQLKYNKIRRDIIPFKCSKNMKSVDEYVNDSEDEELNTKMKNIASRLAPEKYKSVLSQIRDIQIDKESKLVAIMNLLFEKATSDPMLSTAYAYVCRCLTLKRVPSIHCHKEVVNVHKVLNKKCQKEFEKYQVEEAMLNKKLQQIEDTDNNIEKLKFQKELVKDIEIFKRKSQANVRFLCELFKLGVLRENTMQKYIKKMLQSLSENSLESVCLLFNTIGERLDTGKKFKMDVYFIQLKSIAEENSTPNRLKLMIENLISLRENQWNQKEAENHTKQKGITSVKDSMKEETLKVMANSYYENKVKDIYQNGDGSVASNMGDIFSSDPGTKTSSSYQQPQQQQQQQQQHIKEEYVKPGYMKIFDIDAQNKCMDLTQLERKYCNLVYDGTTRASARGDVMVPNCSPNIIRETKEAV

		rna-SPHA_31329	*Sepia esculenta*	MLHQQPNSIEPVTSFATKAAMPSLSPEWKFSPESAPTNIESANRESGPESCFPDAGGIKKYNREFLLQLRHTRASLLMPSCLPDLPRELLKMPYSSKTFNLSCSDPFFKGLKDRRPAEKSRSDESKEGNCFTDSSKMAERYKPLLSEIRCIQIDTESQLKAIIDLLFEKASNYPILGISYAYLCRSLSLIRVPSATRQGETVNFNKLLNRRCQIELEKIKQEDETINQTKQMIRSAEVDETRIIKLQKKLCELTSTSKKRTLGNMKLIGEFFKLHILKENIVIQFVCNLLSTRTEDRIECLCVLLKTVGMELERNSNRQEKKKLEDCFTEMKKIVSQGTCSPRVKSMLSSIIRLRENKWVF
		rna-SPHA_31329	*Sepia officinalis*	MLHQQPNSIEPVTSFATKAATPSLSPEWKFSSESAPTNIESTNRESGPESCFPDPGGIKKYNREFLLQLRHTRASLLMPSCLPDLPRELLKMPYSSKTFNLSCSDPFYKGLKDRRPAEKSRSDESKEGNCFTDSSKMAERYKPLLSEIRCIQIDTESQLKAIIDLLFEKASNYPILGISYAYLCRSLSLIRVPSATRQGETVNFNKLLNRRCQIELEKIKQEDETINQTKQMIRSAEVDETRIIKLQKKLCELTSTSKKRTLGNMKLIGEFFKLRILKENIVIQFVCNLLSTRTEDRIECLCVLLKSVGMELERNSNRQEKKKLEDCFTEMKKIVSQGTCSPRVKSMLSSIIRLRENKWAF
chrZ (sex)	OG0009585	pred2_41298.1	*Architeuthis dux*	MSFLTTDIKALADLLKQEDSDSDGEQQQGSALLGPGHIGGRTKEQETIPSEKDCGQSKEIWNPQEIIEGSEFDSLSDPRPQPEYEILYKQSVTTEDIFLQMGNKTPNTSSCENMVIKIKLPNTELKNITLDVKSIFVDVRTPKYKLGLHLPHPVEEQESNAKWDACQESLIITLKMKREFDFMNY
		Dopeav2130641m.g	*Doryteuthis pealeii*	MGTTKLGSAKFGPGHVGETKKQENISSRKNWALTTQSTVTEENPSAREHSQLHHLSTLSAREINNYFSQSKEIWKPEEIIEGSEFDSLSDPRPQPEYEIFYKQSVTTENIFLQMGNKNPSTSSCENMVIKIKLPNTEMKNITLDIKSVFLDVRTPKYKLGLHLPHPVDEKESNAKWDGCQETLIVTMKMNREFDFLNY*
		g28227.t1	*Euprymna scolopes*	MSFRVSEIAALTNLLTPANEESDSDEDQPQLGSSKFGPGHIGESKKQRNSASKKDINQSKEIWCSEEITEGAEFDSLSDPRLQPELCHSVHYLQGRHKVTVYQ
		LOC_00003860-mRNA-1	*Illex illecebrosus*	MSFMTSDLLALTDLLREPEDSDSDEETRYQGTARLGPGHIGKNQKQETSSSKKDISQSKEIWNPEEINQGSEFDSLSDPRPQPEYEILYKQAVTSEDIYLQMGNKTPNTSSCENMVVKIKLPNTEMKNISLDVKSKFLDLRTPKYKLGLHLPHPVAEQESNAKWDSDCSSLIVTLKMNRELDFMNY
		obimac_0008966.1	*Octopus bimaculoides*	MAFQQCDIRALANLLREPAEDSDSDTDIVCSDYSYGPGHIGPEKNSKDGTAEKDTKQSKDIWSADEIPQGSEFDSLWDQRLQPEYSIVYNQNVRTEDIFLQMGNKTPGSSSCERMVVKIQLPNTMMKDISLDVKPKFLDLRTPKYKLGLHLPFTVKENESQAQWDGGESCLSVSLKMIREYDFVNF
		XP_036367510.1	*Octopus sinensis*	METIVEIAIMETNEESSTTSNKDEDDFFRNITRFWESRSHRSLKSKVQNLVKTWLEAILKAVLTEVAFFWVCGGHSYGPGHVGPEKNSKDGTAEKDTKQSKDIWSADEIPEGSEFDSLWDQRPQPEYSIVYNQNVRTEDIFLQMGNKTPSSSSCERMVVKIQLPNTMMKDISLDVKPKFLDLRTPKYKLGLHLPFTVKEDESQAQWDGGESCLSVSLKMIREFDFVNF
		rna-SPHA_78646	*Sepia esculenta*	MVTKITRTSILEAITFGKNIFYRFYKMSFSVADIRSLTNLLKQPDEDSDSDGEQLYQGSASLGPGNIGESEKQEKPTFKKDLSQSKEIWHPEEINAGSEFDSLSDPRPQPEYEILYKQSVTTEDIFLQMGNKTPNTSSCENMIIKIKLPNTEMKNITLDVKSIFLDVRTPKYKLGLHLPNPVKEQESTAQWHSDQESLIVTLKLNREFDFLNY
		rna-SPHA_78646	*Sepia officinalis*	YEILYKQSVTTEDIFLQMGNKTPNTSSCENMIIKIKLPNTEMKDITLDVKSIFLDVRTPKYKLGLHLPHPVKEQEGAAKWDTDQESLIVNLKLNREFDFMNY*

Table S1.
